# A Testing and Quarantine Algorithm for Individual International Travelers Using Published Data on WHO-Approved Vaccines and Bayes’ Theorem

**DOI:** 10.3390/vaccines10060902

**Published:** 2022-06-06

**Authors:** FuShiuan Whitney Lee, Jamie Wang, C. Jason Wang

**Affiliations:** 1 Center for Policy, Outcomes and Prevention, Department of Pediatrics, School of Medicine, Stanford University, Stanford, CA 94305, USA; fwlee@stanford.edu; 2 College of Letters and Science, University of California, Los Angeles, CA 90095, USA; jamiewang01@gmail.com; 3 Center for Health Policy, Freeman-Spogli Institute for International Studies, Stanford University, Stanford, CA 94305, USA; 4 Department of Health Policy, Stanford University School of Medicine, Stanford, CA 94305, USA

**Keywords:** COVID-19, pandemic, travel policy, testing and quarantine policy, vaccinated travelers

## Abstract

Policies such as border closures and quarantines have been widely used during the COVID-19 pandemic. Policy modifications and updates, however, must be adjusted as global vaccination rates increase. We calculated the risks of individual travelers based on their expected transmission and benchmarked them against that of an unvaccinated traveler quarantined for 14 days without testing. All individuals with a negative preboarding test can be released with a negative arrival test, when both tests have a sensitivity ≥ 90% and a specificity ≥ 97%, performance characteristics that could be accomplished by rapid antigen tests. This assumption is valid for an incidence rate up to 0.1 (prior to testing) and effective reproduction number (Rt) up to 4 in the arrival country. In a sensitivity analysis scenario where the incidence rate is 0.4 and Rt is 16, a negative preboarding test and a negative arrival test, both with a sensitivity ≥ 98% and a specificity ≥ 97%, can ensure that a traveler has a lower expected transmission than an unvaccinated person who is quarantined for 14 days. In most cases, fully vaccinated travelers (with or without booster) and a negative preboarding test can be released with a negative rapid antigen test upon arrival, allowing travelers to depart the airport within 30 min.

## 1. Introduction

Screening for infectious disease among travelers arriving from abroad has been considered useful for preventing transmission of disease in the arrival country [1]. During the COVID-19 pandemic, screening policies for international travelers to test, trace, and isolate COVID-19 cases have been implemented in many countries [2]. The policies, however, varied widely; in general, stricter border control policies help confine community spread (e.g., China), while more relaxed policies may lead to outbreaks originating from international travelers (e.g., United Kingdom).

In addition to testing, tracing, and isolation, universal quarantine has been employed by many countries. Although quarantine can prevent viral spread from abroad, long quarantines after international travel impose a financial burden on individuals, the opportunity cost of time, and mental and emotional stress [3]. High numbers of quarantined individuals also create strain on governments’ tracking systems. As many countries reach high vaccination rates and have readily available testing and masks, governments can formulate border control policies that provide similar protection to quarantine without significantly burdening international travelers.

Science-based decision making is essential to ease restrictions without unnecessarily exposing people to risk of infection. Previously, Wells et al. reported that a 7-day quarantine with testing on exit is needed to achieve a similar probability of post-quarantine transmission for unvaccinated individuals quarantined for 14 days [4]. Models also showed that a three-day or shorter quarantine with antigen testing on exit may be sufficient in many European countries, based on November 2021 data, considering natural immunity, vaccine coverage, and disease burden in these countries [5]. However, the transmission risk of vaccinated and unvaccinated individuals varies greatly, and the effectiveness of different vaccines results in very different breakthrough infection rates (BIR). A model that reflects the transmission risk of individual travelers is needed to provide guidance for policymakers on setting rules that minimize traveler inconvenience and the host country’s economy.

We formulated a testing and quarantine strategy for fully vaccinated (with or without booster) and unvaccinated travelers that is at least as safe as a 14-day quarantine for unvaccinated individuals, using available scientific evidence from the literature, and by applying different effective reproduction numbers (Rt) to model various variants and restrictive policies in the arrival country that might affect Rt. The model provides a coherent way of assessing travelers’ risk of transmission based on their vaccination status and the vaccine types they received. It is sufficiently robust to address changing incidence rates (IR), variant transmission rates, restriction policies, and waning vaccine effectiveness.

## 2. Materials and Methods

To formulate testing and quarantine policies for fully vaccinated travelers (defined as one dose for Janssen and two doses for other vaccines) and unvaccinated travelers, we drew a decision tree addressing the current variant of concern, the Omicron variant, and performed a sensitivity analysis for our model, where our outcome is the expected transmission (E). The expected transmission is defined as the expected number of subsequent infections resulting from an individual, and it is calculated by multiplying the probability of travelers having COVID-19 after one or more negative tests, with Rt of the viral strain/variant. The Rt is dependent on the restriction policies in the arrival country; for example, with the highly infectious Omicron variant, the reported Rt was 1.9 in South Korea, where people are more compliant with protective measures (e.g., social distancing and masking) [6], and 3.7 in the United Kingdom, where social norms are less restrictive [7]. We benchmark the expected transmission of vaccinated scenarios to the expected transmission threshold of an unvaccinated traveler quarantined for 14 days without testing, previously calculated to be 0.005 [4].

### 2.1. Definitions

Vaccine efficacy for preventing infection is one minus the quotient of the breakthrough infection rate (BIR), defined as the percentage of COVID-19 cases among people who have been fully vaccinated, and the percentage of COVID-19 in an unvaccinated placebo group, multiplied by 100%. For example, Pfizer vaccine clinical trial outcomes reported vaccine efficacy of 94.6%, while the BIR is 0.000485 [8]. In our study, we used published BIRs to calculate the probability of travelers having COVID-19 after a negative preboarding test, by applying Fagan’s Nomogram with the negative likelihood ratio (LR-), defined as the quotient of false negative and true negative, dependent on the diagnostic test performance characteristics [9]. We assumed that the risk of contracting COVID-19 on the flight is minimal when only those with a negative test board the plane [10].

### 2.2. Model

Bayes’ Theorem is a mathematical formula used for calculating conditional probabilities, and it describes the probability of an event based on prior knowledge of conditions that might be related to the event.

$$Bayes’\;Theorem:\;P\left(D|T\right)=\frac{P\left(D\right)P(T|D)}{P\left(T\right)},\;if\;P\left(T\right)\neq 0$$

In our case, D is the event of having COVID-19, and T is the event of receiving a negative result. P(D|T), a conditional probability, is the probability of a person having COVID-19 given that the person tests negative. P(D) and P(T) are probabilities of observing D and T, respectively. P(T|D) is the probability that a person tests negative when they have COVID-19. This equation is derived by the definition of conditional probability.

$$P\left(D|T\right)=\frac{P\left(D\cap T\right)}{P\left(T\right)},\;if\;P\left(T\right)\neq 0$$

where $P\left(D\cap T\right)$ is the probability of a person having COVID-19 and testing negative.

$$P\left(T|D\right)=\frac{P\left(D\cap T\right)}{P\left(D\right)},\;if\;P\left(D\right)\neq 0$$

$$P\left(D\cap T\right)=P\left(D\right)P(T|D)$$

Substituting $P\left(D\cap T\right)$ with $P\left(D\right)P(T|D)$ yields Bayes’ Theorem.

To ascertain P(D|T), we must use known information. Known parameters include the prevalence of disease and the sensitivity (the probability of a positive result in a patient with the disease) and specificity (the probably of a negative result in an uninfected individual) of different diagnostic tests. Therefore, sensitivity can be written as P(T’|D), where T’ is an event of receiving a positive result instead of a negative result, and specificity can be written as P(T|D’), where D’ is an event of not having COVID-19.

$$P\left(D|T\right)=\frac{P\left(D\right)P\left(T|D\right)}{P\left(T\right)}\;=\frac{P\left(D\right)P(T|D)}{P\left(D\right)P\left(T|D\right)+P\left(D^{\prime }\right)P(T|D^{\prime })}\;=\frac{\frac{P\left(D\right)P(T|D)}{P\left(D^{\prime }\right)P(T|D^{\prime })}}{\frac{P\left(D\right)P\left(T|D\right)+P\left(D^{\prime }\right)P\left(T|D^{\prime }\right)}{P\left(D^{\prime }\right)P(T|D^{\prime })}}\;=\frac{\frac{P\left(D\right)P(T|D)}{P\left(D^{\prime }\right)P(T|D^{\prime })}}{1+\frac{P\left(D\right)P\left(T|D\right)}{P\left(D^{\prime }\right)P(T|D^{\prime })}}\;=\frac{\frac{P\left(D\right)}{P\left(D^{\prime }\right)}\times \frac{P(T|D)}{P(T|D^{\prime })}}{1+\left[\;\frac{P\left(D\right)}{P\left(D^{\prime }\right)}\times \frac{P\left(T|D\right)}{P\left(T|D^{\prime }\right)}\;\right]}\;\frac{P\left(D\right)}{P\left(D^{\prime }\right)}=\frac{P\left(D\right)}{1-P\left(D\right)}=Odds\;ratio\;of\;having\;the\;disease\;before\;testing\;\left(pretest\;odds\right)\;\frac{P(T|D)}{P(T|D\prime )}=\frac{1-P(T^{\prime }|D)}{P(T|D^{\prime })}=\frac{1-Sensitivity}{Specificity}=LR-$$

By rearranging the equation, we can also conclude that the multiplication of pretest odds and LR- is the posttest odds.

$$P\left(D|T\right)=\frac{\frac{P\left(D\right)}{P\left(D^{\prime }\right)}\times \frac{P(T|D)}{P(T|D^{\prime })}}{1+\left[\;\frac{P\left(D\right)}{P\left(D^{\prime }\right)}\times \frac{P\left(T|D\right)}{P\left(T|D^{\prime }\right)}\;\right]}\;\;\;\;\;\;\;\;\;\;\;\;\;\;\;\;\;\;\;\;\;\;\;\;\;\;\;\;\;\;\;\;\;\;\;\;\;\;\;\;\;\;\;\frac{P\left(D\right)}{P\left(D^{\prime }\right)}\times \frac{P(T|D)}{P(T|D^{\prime })}=\frac{P(D|T)}{1-P(D|T)}\;=Odds\;ratio\;of\;having\;the\;disease\;after\;testing\;\left(posttest\;odds\right)$$

Given the equations, we can summarize the steps to determine posttest probability, P(D|T), the probability of a person having COVID-19 given that the person tests negative. The Fagan’s Nomogram is a graphical tool that simplifies the steps, calculating posttest probability with known pretest probability and likelihood ratio.

1. Calculate pretest odds with disease prevalence (pretest probability):

$$Pretest\;odds=\frac{P\left(D\right)}{1-P\left(D\right)}=\frac{P\left(D\right)}{P\left(D^{\prime }\right)}$$

2. Calculate LR- with sensitivity and specificity:

$$LR-=\frac{1-sensitivity}{specificity}=\frac{P(T|D)}{P(T|D\prime )}$$

3. Multiply pretest odds by LR- and obtain the posttest odds:

$$Posttest\;odds=pretest\;odds\times LR-=\frac{P\left(D\right)}{P\left(D^{\prime }\right)}\times \frac{P(T|D)}{P(T|D^{\prime })}$$

4. Calculate posttest probability with posttest odds:

$$Posttest\;probability=P\left(D|T\right)=\frac{\frac{P\left(D\right)}{P\left(D^{\prime }\right)}\times \frac{P(T|D)}{P(T|D^{\prime })}}{1+\left[\;\frac{P\left(D\right)}{P\left(D^{\prime }\right)}\times \frac{P\left(T|D\right)}{P\left(T|D^{\prime }\right)}\;\right]}$$

In Bayes’ Theorem, the pretest probability is typically the prevalence of the disease. However, for COVID-19, incidence (i.e., the rate of new infection) may be more relevant because people with mild to moderate disease are usually infectious no longer than 10 days after symptom onset [11]. In fully vaccinated travelers, the probability of having COVID-19 is BIR; in unvaccinated travelers, the probability of having COVID-19 is the incidence rate (IR) of COVID-19 in the unvaccinated.

The expected transmission, E, defined as the expected number of subsequent infections resulting from an individual, is calculated by multiplying the probability of travelers having COVID-19 after one or more negative tests, with Rt of the viral strain/variant. If E ≤ 0.005, travelers can be released because their transmission risks are less than or equal to that of an unvaccinated traveler quarantined for 14 days; if E > 0.005, an additional test is warranted [4].

**Figure 1:**
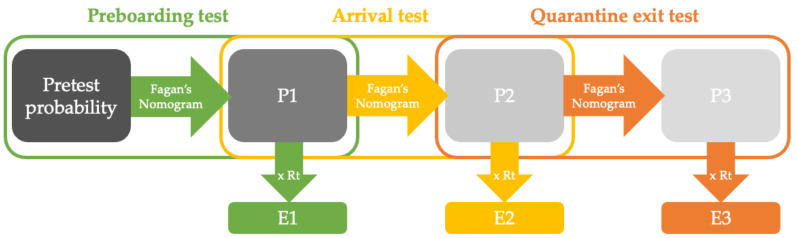
Illustrative summary of the model.

Assuming that the risk of contracting COVID-19 on the flight is minimal following a negative preboarding test, the posttest probability given a negative preboarding test (P1) would be the pretest probability before the arrival test is carried out. Similarly, the posttest probability given a negative arrival test (P2) would be the pretest probability before the quarantine exit test is carried out, if quarantine is needed, and assuming the risk of contracting COVID-19 during quarantine is minimal. We summarized the calculations below and illustrated them in Figure 1.

- P1 is the posttest probability of the preboarding test as well as the pretest probability of the arrival test, assuming that in-flight transmission is minimal when a negative preboarding test is required. P2 is the posttest probability of the arrival test as well as the pretest probability of the quarantine exit test, assuming that the risk of contracting COVID-19 during the quarantine period is minimal. P3 is the posttest probability of the quarantine exit test.
- Effective reproduction number (Rt): The expected number of infections consequent to a single infected individual. Rt depends, in part, on the traveler restriction policies of the arrival country.
- Expected transmission (E): The expected number of subsequent infections resulting from an individual. The expected transmission equals 0.005 in unvaccinated people who quarantined for 14 days. If the expected transmission is less than or equal to 0.005, the traveler can be released into the arrival country. However, if the expected transmission exceeds 0.005, additional test(s) and/or quarantine would be required to safely release the traveler.

1. Expected transmission in travelers
  = Probability of having COVID-19 × Rt
2. Expected transmission in travelers with negative preboarding test, E1
  = Preboarding posttest probability P1 × Rt
  = {[BIR/(1-BIR)] × LR-}/{1+ [BIR/(1-BIR)] × LR-} × Rt
3. Expected transmission in travelers with negative preboarding and arrival tests, E2
  = Arrival posttest probability P2 × Rt
  = {[P1/(1-P1)] × LR-}/{1+ [P1/(1-P1)] × LR-} × Rt
4. Expected transmission in travelers with negative preboarding, arrival, and quarantine exit tests, E3
  = Quarantine exit posttest probability × Rt
  = {[P2/(1-P2)] × LR-}/{1+ [P2/(1-P2)] × LR-} × Rt

### 2.3. Numbers

The BIRs in Table 1 and Table 2 are derived from clinical trials that identified symptomatic breakthrough rates (0.000485 for Pfizer, 0.000778 for Moderna, 0.006081 for AstraZeneca, 0.005996 for Janssen, 0.002040 for Sinopharm, 0.001372 for Sinovac, 0.001425 for Novavax, and 0.002833 for Covaxin) [8,12,13,14,15,16,17,18]. To account for asymptomatic infections, we assumed an equal number of asymptomatic infections (50%). The adjusted BIR, therefore, is twice the BIR rates above. We used this factor because the pooled percentage of asymptomatic infections among the confirmed population is 40.5% (95% CI, 33.5%–47.5%) [19]. The resulting adjusted BIRs range from 0.00097 to 0.01216. Further, we multiplied the adjusted BIRs by ten, to account for current variants of concern and waning vaccine effectiveness. This yielded the highest 20× BIR of 0.1216. To provide a general policy for all vaccines, we chose 0.13 as the highest possible BIR for all World Health Organization (WHO)-approved vaccines.

In Table 3, we assumed 0.5 to be the IR of COVID-19 among the unvaccinated. According to the United States Centers for Disease Control and Prevention (CDC), in December 2021, unvaccinated individuals had 2.8 times the age-standardized case incidence rate compared to fully vaccinated people without a booster shot [20]. As we modeled the general policy for vaccinated travelers with a BIR (IR among the 2-dose vaccinated) of 0.13, the IR of the unvaccinated would be approximately 0.364. To be more conservative and account for unreported COVID-19 cases, we modeled Table 3 with an IR of 0.5 among the unvaccinated travelers. In addition, the modeled IR is greater than the probability of becoming infected among individuals who are household members of COVID-19-positive cases, which is reported to be 0.427 for the Omicron variant, 0.297 for the Delta variant, 0.364 for the Alpha variant, and 0.189 for the wild-type variant [21].

To account for different levels of restrictions across countries, we used Rt < 3 and Rt < 10 to model for policy options in countries with strict restrictions and loose restrictions, respectively (Table 1, Table 2 and Table 3).

In Table 4, we report the sensitivity analysis of our model. We calculated the expected transmissions considering various IRs and Rts, with IR up to 0.5 and Rt up to 32. The IR for vaccinated people is the BIR.

We split diagnostic tests into three different categories: PCR, tests with a sensitivity ≥ 98% and a specificity ≥ 97%, usually achieved by using the Polymerase Chain Reaction (PCR)/Nucleic Acid Amplification (NAAT) tests, rapid tests with higher sensitivity (RPD *): any test with a sensitivity ≥ 90% and a specificity ≥ 97%, and rapid tests (RPD): any test with a sensitivity ≥ 80% and a specificity ≥ 97%, based on recommendations from the WHO [22].

### 2.4. Example

In a traveler who received two doses of the Sinovac vaccine, the probability of having COVID-19 (adjusted BIR) is 0.027443, accounting for asymptomatic infection, variants of concern, and waning vaccine effectiveness. The pretest odds would be 0.027443/(1 − 0.027443) = 0.028218. If the traveler tested negative with a preboarding PCR test, the posttest odds of having COVID-19 would be 0.028218 × (1 − 98%)/97% = 0.000582. The posttest probability would then be 0.000582/(1 + 0.000582) = 0.000581. Accordingly, the expected transmission with Rt = 10 would be 0.000581 × 10 = 0.00581. As 0.00581 is higher than 0.005, the traveler would need an arrival test. The pretest probability of the arrival test would be the posttest probability of the preboarding test, which is 0.000581. The pretest odds of the arrival test would be 0.000581/(1 − 0.000581) = 0.000582, the posttest odds would be 0.000582 × (1 − 80%)/97% = 0.00012 if the arrival RPD test reported negative, and the expected transmission with Rt = 10 would be 0.00012 × 10 = 0.0012. As 0.0012 is less than 0.005, the traveler can be released from the airport.

## 3. Results

### 3.1. Model for Vaccinated and Unvaccinated Travelers

The decision tree in Table 1 describes testing and quarantine strategies for travelers fully vaccinated without a booster, regardless of vaccine type (using 0.13 as the highest possible BIR for all WHO-approved vaccines). To release fully vaccinated travelers with a negative preboarding test with a sensitivity ≥ 90% and a specificity ≥ 97% (RPD *) into an arrival country that has low vaccination rates and loose restriction policies (assumed Rt < 10), an arrival test with a sensitivity ≥ 98% and a specificity ≥ 97% (PCR) would be required to be valid for all WHO-approved vaccines. However, if the preboarding test turns out to be unacceptable, these travelers should have a negative arrival test with a sensitivity ≥ 98% and a specificity ≥ 97% (PCR), quarantined for four days, and be released if the post-quarantine test with a sensitivity ≥ 90% and a specificity ≥ 97% (RPD *) is negative. The rationale for a four-day quarantine for individuals without valid preboarding tests is that the current dominant Omicron variant has an incubation period of 72 hours, and serial testing during this period should allow detection of the virus [23].

Assumptions (see main text and Figure 1 for rationale and detailed calculations):

- Effective reproduction number (Rt): The expected number of infections consequent to a single infected individual. We assume that countries with strict restrictive policies have Rt < 3, and countries with loose restrictive policies have Rt < 10.
- Expected transmission (E): The expected number of subsequent infections resulting from an individual. The expected transmission equals to 0.005 in unvaccinated people who quarantined for 14 days. If the expected transmission is less than or equal to 0.005, the traveler can be released into the arrival country. However, if the expected transmission is greater than 0.005, he/she will need additional test(s) and/or quarantine to be released.
- The probability of contracting COVID-19 in fully vaccinated (with or without booster) individuals prior to testing is assumed to be 0.13 in the model. The highest breakthrough infection rate (BIR) among the WHO-approved vaccines reported in the clinical trial is 0.01216. We multiplied the BIR by two to account for asymptomatic infections, and further multiplied it by ten to account for current variants of concern and waning vaccine effectiveness. This yielded the highest 20× BIR of 0.1216. Therefore, to provide a general policy for all vaccines, we chose 0.13 as the highest possible BIR for all WHO-approved vaccines.

Caveat: Travelers without a preboarding test can refer to the “Unacceptable” rows.

Table 2 describes the strategy when individual vaccines are considered. In arrival countries with strict restriction policies (e.g., mask wearing, social distancing) in place (assumed Rt < 3), travelers vaccinated with Pfizer or Moderna can be released on arrival if they had a negative preboarding test with a minimum sensitivity of 90% and a minimum specificity of 97% (RPD *). Travelers vaccinated with Sinopharm, Sinovac, Novavax, or Covaxin would require an additional negative arrival test with a sensitivity ≥ 80% and a specificity ≥ 97% (RPD) to be released, while travelers vaccinated with AstraZeneca or Janssen would require a more sensitive arrival test with a sensitivity ≥ 90% and a specificity ≥ 97% (RPD *) that is negative to be released.

Assumptions (see main text and Figure 1 for rationale and detailed calculations):

- Adjusted breakthrough infection rate (BIR): The probability of a fully vaccinated individual getting COVID-19. The adjusted BIRs are two times higher than the BIRs of the clinical trials, accounting for the assumed 50% asymptomatic breakthrough infection cases. We used 10 times the adjusted BIRs to account for current variants of concern and waning vaccine effectiveness.
- Effective reproduction number (Rt): The expected number of infections consequent to a single infected individual. We assume that countries with strict restrictive policies have Rt < 3, and countries with loose restrictive policies have Rt < 10.
- The expected transmission (expected number of subsequent infections resulting from an individual) equals 0.005 in unvaccinated people who quarantined for 14 days. If the expected transmission is less than or equal to 0.005, the traveler can be released into the arrival country. However, if the expected transmission is greater than 0.005, he/she will need additional test(s) and/or quarantine to be released.

Caveat: Sinovac, Sinopharm, Novavax, and Covaxin have not reported their rates of waning efficacy after six months. Once their efficacies are known, policies targeting these vaccines may need to be adjusted (Table 4).

In Table 3, we describe the strategy for unvaccinated travelers. When unvaccinated travelers arrive in countries with strict restriction policies (R < 3), they can be released with a negative arrival test with a sensitivity ≥ 98% and a specificity ≥ 97% (PCR) if they had a negative preboarding PCR test. In situations where unvaccinated travelers presented with a preboarding test with a sensitivity ≥ 80% and a specificity ≥ 97% (RPD) at an arrival country with a loose restriction policy (R < 10), they can be released after quarantining for four days with two negative tests: an arrival test with a sensitivity ≥ 98% and a specificity ≥ 97% (PCR) on quarantine day 1, and a quarantine exit test with a sensitivity ≥ 80% and a specificity ≥ 97% (RPD) on quarantine day 4.

Assumptions (see main text and Figure 1 for rationale and detailed calculations):

- Effective reproduction number (Rt): The expected number of infections consequent to a single infected individual. We assume that countries with strict restrictive policies have Rt < 3, and countries with loose restrictive policies have Rt < 10.
- Expected transmission (E): The expected number of subsequent infections resulting from an individual. The expected transmission equals 0.005 in unvaccinated people who quarantined for 14 days. If Ex is less than or equal to 0.005, the traveler can be released into the arrival country. However, if Ex is greater than 0.005, he/she will need additional test(s) and/or quarantine to be released.
- The probability of contracting COVID-19 in unvaccinated individuals prior to testing is assumed to be 0.5 in the model. The probability of contracting COVID-19 in fully vaccinated individuals prior to testing is assumed to be 0.13 (highest possible BIR for all WHO-approved vaccines) in Table 1. According to the CDC, the age-standardized case incidence rate ratio during December 2021 (peak of Omicron variant wave) was 2.8 comparing unvaccinated people with fully vaccinated people without a booster shot. Therefore, the incidence rate of the unvaccinated would be approximately 0.364. To be more conservative and account for COVID-19 cases that did not get tested, we modeled Table 3 with an incidence rate of 0.5 in the unvaccinated travelers.

### 3.2. Sensitivity Analysis of Our Model

Table 4 shows the interventions needed for an individual to be released using the expected transmission threshold of 0.005, accounting for variants with different transmissibility (presented with Rt of 1, 2, 4, 8, 16, and 32) and possible IRs for vaccinated and unvaccinated individuals (0.01, 0.05, 0.1, 0.15, 0.2, 0.25, 0.3, 0.35, 0.4, 0.45, and 0.5).

The cell color in Table 4 indicates the minimum intervention needed: direct release (green), arrival RPD test (light yellow), arrival RPD * test (yellow), arrival PCR test (dark yellow), arrival PCR test plus quarantine and RPD test before release (light orange), arrival PCR test plus quarantine and RPD * test before release (orange), arrival PCR test plus quarantine and PCR test before release (dark orange), and arrival PCR test plus quarantine and two tests with PCR and RPD before release (red).

For example, the Omicron variant has been reported to spread faster and infect more vaccinated people (higher Rt and BIR). If the IR is assumed to be 1 in 20 (0.05), and Rt with restriction policies in place is estimated to be 4, travelers with a negative preboarding test with a sensitivity ≥ 98% and a specificity ≥ 97% (PCR) can be released directly (Table 4a). If the preboarding test has a sensitivity between 98% and 90%, and a specificity above 97%, an additional negative arrival test with a sensitivity ≥ 80% and a specificity ≥ 97% (RPD) can ensure that the released traveler possesses a lower risk of transmitting COVID-19 than an unvaccinated traveler quarantined for 14 days (Table 4b).

Table 4a shows that even with a variant that has transmissibility of Rt = 8 and infects 1 in 5 people (IR = 0.2), a traveler with a negative preboarding PCR test would only need a negative arrival RPD * test to be released. However, with the same variant dominating, travelers without an acceptable preboarding test would need to take an arrival PCR test, quarantine for four days, and receive an RPD * test before release (Table 4d).

## 4. Discussion

We provided an algorithm that predicts the transmission risk of individual travelers based on their vaccination status and the type of vaccine they received. Our model shows that all individuals with a negative preboarding test can be released with a negative arrival test, with both tests having a sensitivity ≥ 90% and a specificity ≥ 97%, even when IR reaches 0.1 and Rt is 4. Rapid tests meet these performance characteristics and can be performed easily and quickly, allowing travelers with negative test results to depart the airport within 30 minutes. This flexible model can be used for different viral variants, vaccine effectiveness, and restriction policies, and is therefore adaptable internationally.

A Qatari pilot program from February to April 2021 is a test case for our model, where quarantine requirements were waived for vaccinated residents who received their second mRNA vaccine dose (99.7% Pfizer) at least 14 days before arrival [24]. No consideration was given to preboarding test status. On arrival, each individual was tested using a PCR test: 83 out of the 10,092 fully vaccinated people tested positive, with a BIR of 0.0082 (8.5 times the adjusted BIR of the Pfizer clinical trial). The scenario aligns with the algorithm that we used for travelers vaccinated with the Pfizer vaccine without a preboarding test, that requires a negative PCR test before release (Table 2). This is what Qatar has implemented for arriving passengers.

In Table 1, Table 2 and Table 3, we assumed Rt < 3 in countries with strict restriction policies, with empirical evidence that Rt was 2.56 (2.23, 2.96) in South Africa in December 2021 when the Omicron variant dominated [25]. For countries with fewer restrictions, the tables provide Rt of 10 as the upper limit. When Rt > 10, we believe governments must implement some restrictions to flatten the curve. In addition, we modeled Table 1 and Table 2 with ten times the adjusted BIR of WHO-approved vaccines (20 times the BIR from clinical trials) to account for waning effects of the vaccines and variants of concern. When the Omicron variant dominated the reported infections in the United States, the age-adjusted 14-day cumulative incidence was 3355.5 per 100,000 in fully vaccinated people and 6743.5 per 100,000 in unvaccinated people [26]. Accordingly, the BIR is estimated to be 0.0336 in fully vaccinated people and the IR is 0.0674 in unvaccinated people. Therefore, Table 1, Table 2 and Table 3 are still valid with the Omicron variant being the dominant variant.

There are extensions to our study. First, we suggest that partially vaccinated individuals should be treated as unvaccinated individuals since the BIRs for partially vaccinated individuals vary greatly in the first three weeks following the initial dose [27]. Second, governments can use Table 4 to extrapolate testing and quarantine strategies for the vaccines not listed in the tables. For example, when formulating policies for travelers vaccinated with Sputnik V, policymakers can refer to IR = 0.05 in Table 4 because it is greater than 0.02138, which is 20 times the reported clinical trial BIR of Sputnik V, accounting for asymptomatic infections and waning vaccine efficacy [28]. Third, travelers vaccinated with mixed vaccines can follow the policy for the least effective vaccine in the mix. For example, studies have shown that a dose of AstraZeneca followed by an mRNA vaccine creates antibody responses higher than two doses of AstraZeneca [29]; as such, the AstraZeneca branch of Table 2 may overestimate expected transmission but can be used for travelers receiving these mix-and-match vaccines. Fourth, governments can extrapolate testing and quarantine strategies for unvaccinated individuals with confirmed prior COVID-19 infection. Reinfection rate, a proxy for BIR for natural immunity, in the Omicron variant-dominant period is 0.027 [30]; as such, policymakers can reference rows IR = 0.05 in Table 4. If restriction policies are in place and Rt in the country is under 2, unvaccinated travelers with proof of prior infection can be released if they received negative results in both preboarding and arrival tests with a sensitivity ≥ 80% and a specificity ≥ 97%.

If we account for known waning immunity of the vaccines, the decision tree (Table 2) could still be valid for Pfizer, Moderna, Janssen, and AstraZeneca up to six months after the second dose. The BIR of people fully vaccinated with Pfizer after six months is 0.0035 in Israel [31], less than ten times the adjusted BIR (0.0097) modeled for Pfizer in Table 2. Even though Moderna, Janssen, and AstraZeneca have not reported BIR six months after the second dose, when compared to Pfizer, studies of these vaccines have shown a slower decline in vaccine effectiveness five to six months after the second dose [32]. However, Sinovac, Sinopharm, Novavax, and Covaxin have not reported their rates of waning efficacy after six months. Once extended efficacies are known, policies targeting these vaccines may need to be adjusted (Table 4).

Boosters are important in mitigating the waning effects of vaccines and can provide additional protection. For example, travelers vaccinated with three doses of Pfizer or AstraZeneca have vaccine efficacies above 93%, similar to or better than the original clinical trials with Pfizer and AstraZeneca [33]. Additionally, booster vaccines can result in antibody levels higher than the original two doses, e.g., Moderna showed higher neutralization titers 28 days following a booster compared to 28 days following the second dose [34]. The seroconversion rates of neutralizing antibodies toward COVID-19 variants were higher in people vaccinated with three Sinovac doses compared to two [35]. Further, early antibody level data suggest that boosters may further protect against the Omicron variant [35,36]. With preliminary data suggesting that three doses of Pfizer produce an immune response against the Omicron variant similar to that of two doses against earlier variants, governments may consider a three-shot requirement for travelers to qualify as fully vaccinated.

Our model is flexible and can be adapted to real-life situations. For example, when in doubt, governments can choose to not consider the information provided by the negative preboarding test at all—and refer to the “Unacceptable” rows in Table 1, Table 2 and Table 3. When establishing the requirements for the type of test and the timeframe for testing, we suggest that governments establish rules based on real-world conditions to ensure that the results can be trusted. For example, a policy that requires negative PCR tests collected within 48 hours prior to boarding is logistically impossible in countries unable to provide accessible, fast, and affordable PCR tests. This may lead to false reporting. Conversely, a policy that only requires rapid antigen testing within 24 hours could be feasible while accomplishing the same protections from transmission.

## 5. Limitations

The data from the randomized controlled clinical trials we reference may not be identical to the real-world data (Table 2). For example, Sinovac and Sinopharm reported lower BIR compared to AstraZeneca; however, their effectiveness is less certain because they provided lower antibody responses compared to AstraZeneca and severe outbreaks were observed in countries mainly vaccinated with the two vaccines, despite rollout success [37]. Real-world BIRs are needed to provide additional evidence for decision making.

Although we have modeled for extreme scenarios where the IR of COVID-19 reaches 0.5 and the Rt reaches 32, it is still possible that a new variant during the pandemic can exceed the limits of what we modeled. Additional calculations can be performed to expand on the model.

We assumed that in-flight transmission is minimal if a preboarding test is used. However, it is possible that travelers can contract COVID-19 after the preboarding test or become exposed to the virus during the flight. These travelers may receive negative results in the arrival test because the arrival test is performed too soon following infection. To prevent viral spread from infected travelers with negative results, governments can choose to require testing results three days after travelers are released from the airport and isolate the positive cases.

Future research is needed to further validate our model with government-collected data on travelers’ vaccination status, type of vaccination, Rt, and BIR or IR among the vaccinated and unvaccinated travelers.

## 6. Conclusions

Our test and release strategy is evidence-based and applicable for different vaccination statuses, vaccine types, and testing options. This approach can be time- and cost-saving. For those who are financially vulnerable (e.g., migrant workers), and those with time-sensitive issues (e.g., business travelers), this policy could avoid quarantine except in situations where travelers are infected, or transmission risk is high.

## Figures and Tables

**Table 1:** Testing and quarantine strategies for fully vaccinated travelers (general).

Preboarding Test Type	E1, if Tested Negative	Policy	Arrival Test Type	E2, if Tested Negative	Policy	Quarantine Exit Test Type	E3, if Tested Negative	Policy
**Rt < 3**								
PCR	0.0092	Test	PCR	0.0002	Release			
			RPD *	0.0010	Release			
			RPD	0.0019	Release			
RPD *	0.0455	Test	PCR	0.0010	Release			
			RPD *	0.0048	Release			
			RPD	0.0095	Quarantine	RPD	0.0020	Release
RPD	0.0897	Test	PCR	0.0019	Release			
			RPD *	0.0095	Quarantine	RPD	0.0020	Release
			RPD	0.0189	Quarantine	RPD	0.0039	Release
Unacceptable	0.3900	Test	PCR	0.0092	Quarantine	RPD	0.0019	Release
			RPD *	0.0455	Quarantine	RPD *	0.0048	Release
			RPD	0.0897	Quarantine	PCR	0.0019	Release
**Rt < 10**								
PCR	0.0307	Test	PCR	0.0006	Release			
			RPD *	0.0032	Release			
			RPD	0.0063	Quarantine	RPD	0.0013	Release
RPD *	0.1517	Test	PCR	0.0032	Release			
			RPD *	0.0159	Quarantine	RPD	0.0033	Release
			RPD	0.0317	Quarantine	RPD *	0.0033	Release
RPD	0.2989	Test	PCR	0.0063	Quarantine	RPD	0.0013	Release
			RPD *	0.0317	Quarantine	RPD *	0.0033	Release
			RPD	0.0631	Quarantine	PCR	0.0013	Release
Unacceptable	1.3000	Test	PCR	0.0307	Quarantine	RPD *	0.0032	Release
			RPD *	0.1517	Quarantine	PCR	0.0032	Release
			RPD	0.2989	Quarantine	PCR	0.0063	Test with RPD

Abbreviations: PCR: Any test that meets the minimum requirement of sensitivity ≥ 98% and specificity ≥ 97%, achieved by most Nucleic Acid Amplification tests (NAAT). RPD *: Any test that meets the minimum requirement of sensitivity ≥ 90% and specificity ≥ 97%, achieved by many rapid antigen tests. RPD: Any test that meets the minimum requirement of sensitivity ≥ 80% and specificity ≥ 97% (WHO recommendation for COVID-19 diagnostic tests).

**Table 2:** Testing and quarantine strategies for fully vaccinated travelers (individual vaccines).

Rt < 3					Rt < 10				
Vaccine Type (Adjusted BIR ×10)	Preboarding Test	Policy if Test Negative	Arrival Test	Policy if Test Negative	Vaccine Type (Adjusted BIR ×10)	Preboarding Test	Policy if Test Negative	Arrival Test	Policy if Test Negative
**Pfizer** (0.0097)	PCR	Release			**Pfizer** (0.0097)	PCR	Release		
	RPD *	Release				RPD *	Test	PCR	Release
								RPD *	Release
								RPD	Release
	RPD	Test	PCR	Release		RPD	Test	PCR	Release
			RPD *	Release				RPD *	Release
			RPD	Release				RPD	Release
	Unacceptable	Test	PCR	Release		Unacceptable	Test	PCR	Release
			RPD *	Release				RPD *	Quarantine ^a^
			RPD	Quarantine ^a^				RPD	Quarantine ^a^
**Moderna** (0.0156)	PCR	Release			**Moderna** (0.0156)	PCR	Release		
	RPD *	Release				RPD *	Test	PCR	Release
								RPD *	Release
								RPD	Release
	RPD	Test	PCR	Release		RPD	Test	PCR	Release
			RPD *	Release				RPD *	Release
			RPD	Release				RPD	Quarantine ^a^
	Unacceptable	Test	PCR	Release		Unacceptable	Test	PCR	Release
			RPD *	Release				RPD *	Quarantine ^a^
			RPD	Quarantine ^a^				RPD	Quarantine ^b^
**AstraZeneca** (0.1216)	PCR	Test	PCR	Release	**AstraZeneca** (0.1216)	PCR	Test	PCR	Release
			RPD *	Release				RPD *	Release
			RPD	Release				RPD	Quarantine ^a^
	RPD *	Test	PCR	Release		RPD *	Test	PCR	Release
			RPD *	Release				RPD *	Quarantine ^a^
			RPD	Quarantine ^a^				RPD	Quarantine ^b^
	RPD	Test	PCR	Release		RPD	Test	PCR	Quarantine ^a^
			RPD *	Quarantine ^a^				RPD *	Quarantine ^b^
			RPD	Quarantine ^a^				RPD	Quarantine ^c^
	Unacceptable	Test	PCR	Quarantine ^a^		Unacceptable	Test	PCR	Quarantine ^b^
			RPD *	Quarantine ^b^				RPD *	Quarantine ^c^
			RPD	Quarantine ^c^				RPD	Quarantine ^d^
**Janssen** (0.1199)	PCR	Test	PCR	Release	**Janssen** (0.1199)	PCR	Test	PCR	Release
			RPD *	Release				RPD *	Release
			RPD	Release				RPD	Quarantine ^a^
	RPD *	Test	PCR	Release		RPD *	Test	PCR	Release
			RPD *	Release				RPD *	Quarantine ^a^
			RPD	Quarantine ^a^				RPD	Quarantine ^b^
	RPD	Test	PCR	Release		RPD	Test	PCR	Quarantine ^a^
			RPD *	Quarantine ^a^				RPD *	Quarantine ^b^
			RPD	Quarantine ^a^				RPD	Quarantine ^c^
	Unacceptable	Test	PCR	Quarantine ^a^		Unacceptable	Test	PCR	Quarantine ^b^
			RPD *	Quarantine ^b^				RPD *	Quarantine ^c^
			RPD	Quarantine ^c^				RPD	Quarantine ^d^
**Sinopharm** (0.0408)	PCR	Release			**Sinopharm** (0.0408)	PCR	Test	PCR	Release
								RPD *	Release
								RPD	Release
	RPD *	Test	PCR	Release		RPD *	Test	PCR	Release
			RPD *	Release				RPD *	Release
			RPD	Release				RPD	Quarantine ^a^
	RPD	Test	PCR	Release		RPD	Test	PCR	Release
			RPD *	Release				RPD *	Quarantine ^a^
			RPD	Quarantine ^a^				RPD	Quarantine ^a^
	Unacceptable	Test	PCR	Release		Unacceptable	Test	PCR	Quarantine ^a^
			RPD *	Quarantine ^a^				RPD *	Quarantine ^b^
			RPD	Quarantine ^b^				RPD	Quarantine ^c^
**Sinovac** (0.0274)	PCR	Release			**Sinovac** (0.0274)	PCR	Test	PCR	Release
								RPD *	Release
								RPD	Release
	RPD *	Test	PCR	Release		RPD *	Test	PCR	Release
			RPD *	Release				RPD *	Release
			RPD	Release				RPD	Quarantine ^a^
	RPD	Test	PCR	Release		RPD	Test	PCR	Release
			RPD *	Release				RPD *	Quarantine ^a^
			RPD	Release				RPD	Quarantine ^a^
	Unacceptable	Test	PCR	Release		Unacceptable	Test	PCR	Quarantine ^a^
			RPD *	Quarantine ^a^				RPD *	Quarantine ^b^
			RPD	Quarantine ^a^				RPD	Quarantine ^c^
**Novavax** (0.0285)	PCR	Release			**Novavax** (0.0285)	PCR	Test	PCR	Release
								RPD *	Release
								RPD	Release
	RPD *	Test	PCR	Release		RPD *	Test	PCR	Release
			RPD *	Release				RPD *	Release
			RPD	Release				RPD	Quarantine ^a^
	RPD	Test	PCR	Release		RPD	Test	PCR	Release
			RPD *	Release				RPD *	Quarantine ^a^
			RPD	Release				RPD	Quarantine ^a^
	Unacceptable	Test	PCR	Release		Unacceptable	Test	PCR	Quarantine ^a^
			RPD *	Quarantine ^a^				RPD *	Quarantine ^b^
			RPD	Quarantine ^a^				RPD	Quarantine ^c^
**Covaxin** (0.0567)	PCR	Release			**Covaxin** (0.0567)	PCR	Test	PCR	Release
								RPD *	Release
								RPD	Release
	RPD *	Test	PCR	Release		RPD *	Test	PCR	Release
			RPD *	Release				RPD *	Quarantine ^a^
			RPD	Release				RPD	Quarantine ^a^
	RPD	Test	PCR	Release		RPD	Test	PCR	Release
			RPD *	Release				RPD *	Quarantine ^a^
			RPD	Quarantine ^a^				RPD	Quarantine ^b^
	Unacceptable	Test	PCR	Release		Unacceptable	Test	PCR	Quarantine ^a^
			RPD *	Quarantine ^a^				RPD *	Quarantine ^c^
			RPD	Quarantine ^b^				RPD	Quarantine ^c^

^a^ Quarantine for 4 days, release with negative exit RPD test. ^b^ Quarantine for 4 days, release with negative exit RPD * test. ^c^ Quarantine for 4 days, release with negative exit PCR test. ^d^ Quarantine for 4 days, requires negative exit PCR test on day 3, and an additional negative exit RPD test on day 4 to be released. Abbreviations: PCR: Any test that meets the minimum requirement of sensitivity ≥ 98%, specificity ≥ 97%. RPD *: Any test that meets the minimum requirement of sensitivity ≥ 90%, specificity ≥ 97%. RPD: Any test that meets the minimum requirement of sensitivity ≥ 80%, specificity ≥ 97%.

**Table 3:** Testing and quarantine strategies for unvaccinated travelers.

Preboarding Test Type	E1, if Tested Negative	Policy	Arrival Test Type	E2, if Tested Negative	Policy	Quarantine Test Type on Day 3	E3, if Tested Negative	Policy
**Rt < 3**								
PCR	0.0606	Test	PCR	0.0013	Release			
			RPD *	0.0064	Quarantine	PCR	0.0001	Release
						RPD *	0.0007	Release
						RPD	0.0013	Release
			RPD	0.0127	Quarantine	PCR	0.0003	Release
						RPD *	0.0013	Release
						RPD	0.0026	Release
RPD *	0.2804	Test	PCR	0.0064	Quarantine	PCR	0.0001	Release
						RPD *	0.0007	Release
						RPD	0.0013	Release
			RPD *	0.0315	Quarantine	PCR	0.0007	Release
						RPD *	0.0033	Release
						RPD	0.0066	Test ^a^
			RPD	0.0624	Quarantine	PCR	0.0013	Release
						RPD *	0.0066	Test ^a^
						RPD	0.0131	Test ^a^
RPD	0.5128	Test	PCR	0.0127	Quarantine	PCR	0.0003	Release
						RPD *	0.0013	Release
						RPD	0.0026	Release
			RPD *	0.0624	Quarantine	PCR	0.0013	Release
						RPD *	0.0066	Test ^a^
						RPD	0.0131	Test ^a^
			RPD	0.1223	Quarantine	PCR	0.0026	Release
						RPD *	0.0131	Test ^a^
						RPD	0.0261	Test ^b^
Unacceptable	1.5000	Test	PCR	0.0606	Quarantine	PCR	0.0013	Release
						RPD *	0.0064	Test ^a^
						RPD	0.0127	Test ^a^
			RPD *	0.2804	Quarantine	PCR	0.0064	Test ^a^
						RPD *	0.0315	Test ^b^
						RPD	0.0624	Test ^c^
			RPD	0.5128	Quarantine	PCR	0.0127	Test ^a^
						RPD *	0.0624	Test ^c^
						RPD	0.1223	Test ^c^
**Preboarding Test Type**	**E_1_, if Tested Negative**	**Policy**	**Arrival Test Type**	**E_2_, if Tested Negative**	**Policy**	**Quarantine Test Type on Day 3**	**E_3_, if Tested Negative**	**Policy**
**Rt < 10**								
PCR	0.2020	Test	PCR	0.0042	Quarantine	PCR	0.0001	Release
						RPD *	0.0004	Release
						RPD	0.0009	Release
			RPD *	0.0212	Quarantine	PCR	0.0004	Release
						RPD *	0.0022	Release
						RPD	0.0044	Release
			RPD	0.0423	Quarantine	PCR	0.0009	Release
						RPD *	0.0044	Release
						RPD	0.0088	Test ^a^
RPD *	0.9346	Test	PCR	0.0212	Quarantine	PCR	0.0004	Release
						RPD *	0.0022	Release
						RPD	0.0044	Release
			RPD *	0.1052	Quarantine	PCR	0.0022	Release
						RPD *	0.0109	Test ^a^
						RPD	0.0219	Test ^a^
			RPD	0.2081	Quarantine	PCR	0.0044	Release
						RPD *	0.0219	Test ^a^
						RPD	0.0436	Test ^b^
RPD	1.7094	Test	PCR	0.0423	Quarantine	PCR	0.0009	Release
						RPD *	0.0044	Release
						RPD	0.0088	Test ^a^
			RPD *	0.2081	Quarantine	PCR	0.0044	Release
						RPD *	0.0219	Test ^a^
						RPD	0.0436	Test ^b^
			RPD	0.4078	Quarantine	PCR	0.0088	Test ^a^
						RPD *	0.0436	Test ^b^
						RPD	0.0869	Test ^c^
Unacceptable	5.0000	Test	PCR	0.2020	Quarantine	PCR	0.0042	Release
						RPD *	0.0212	Test ^b^
						RPD	0.0423	Test ^b^
			RPD *	0.9346	Quarantine	PCR	0.0212	Test ^b^
						RPD *	0.1052	Test ^c^
						RPD	0.2081	Test ^c^
			RPD	1.7094	Quarantine	PCR	0.0423	Test ^b^
						RPD *	0.2081	Test ^c^
						RPD	0.4078	Test ^d^

^a^ Release with an additional negative RPD test. ^b^ Release with an additional negative RPD * test. ^c^ Release with an additional negative PCR test. ^d^ Release with an additional negative test of sensitivity ≥ 99%, specificity ≥ 97%. Abbreviations: PCR: Any test that meets the minimum requirement of sensitivity ≥ 98%, specificity ≥ 97%. RPD *: Any test that meets the minimum requirement of sensitivity ≥ 90%, specificity ≥ 97%. RPD: Any test that meets the minimum requirement of sensitivity ≥ 80%, specificity ≥ 97%.

**Table 4:** Regardless of vaccine status, travelers’ expected transmission with negative preboarding, with/without arrival, with/without post-quarantine tests. (**a**) Expected transmission for travelers with a negative preboarding PCR test and a negative arrival test. (**b**) Expected transmission for travelers with a negative preboarding RPD * test and a negative arrival test. (**c**) Expected transmission for travelers with a negative preboarding RPD test and a negative arrival test. (**d**) Expected transmission for travelers with an invalid preboarding test and a negative arrival test.

(a)						
IR	Rt = 1	Rt = 2	Rt = 4	Rt = 8	Rt = 16	Rt = 32
0.01	0.0002	0.0004	0.0008	0.0017	0.0033	0.0014
0.05	0.0011	0.0022	0.0043	0.0018	0.0036	0.0036
0.10	0.0023	0.0046	0.0019	0.0038	0.0038	0.0015
0.15	0.0036	0.0015	0.0030	0.0030	0.0012	0.0024
0.20	0.0011	0.0021	0.0042	0.0042	0.0017	0.0034
0.25	0.0014	0.0028	0.0028	0.0011	0.0023	0.0045
0.30	0.0018	0.0036	0.0036	0.0015	0.0029	0.0012
0.35	0.0023	0.0046	0.0046	0.0018	0.0037	0.0015
0.40	0.0028	0.0028	0.0011	0.0023	0.0045	0.0019
0.45	0.0035	0.0035	0.0014	0.0028	0.0011	0.0023
0.50	0.0042	0.0042	0.0017	0.0034	0.0014	0.0028
(**b**)						
IR	Rt = 1	Rt = 2	Rt = 4	Rt = 8	Rt = 16	Rt = 32
0.01	0.0010	0.0021	0.0042	0.0017	0.0034	0.0034
0.05	0.0011	0.0022	0.0045	0.0045	0.0018	0.0036
0.10	0.0024	0.0047	0.0047	0.0019	0.0038	0.0016
0.15	0.0037	0.0037	0.0015	0.0030	0.0012	0.0025
0.20	0.0026	0.0011	0.0021	0.0042	0.0018	0.0035
0.25	0.0035	0.0014	0.0028	0.0012	0.0023	0.0047
0.30	0.0045	0.0018	0.0036	0.0015	0.0030	0.0030
0.35	0.0011	0.0023	0.0046	0.0019	0.0038	0.0038
0.40	0.0014	0.0028	0.0012	0.0023	0.0047	0.0047
0.45	0.0017	0.0035	0.0014	0.0029	0.0029	0.0011
0.50	0.0021	0.0042	0.0018	0.0035	0.0035	0.0014
(**c**)						
IR	Rt = 1	Rt = 2	Rt = 4	Rt = 8	Rt = 16	Rt = 32
0.01	0.0021	0.0042	0.0017	0.0034	0.0034	0.0014
0.05	0.0022	0.0045	0.0045	0.0018	0.0036	0.0015
0.10	0.0047	0.0047	0.0019	0.0038	0.0016	0.0031
0.15	0.0037	0.0015	0.0030	0.0012	0.0025	0.0049
0.20	0.0011	0.0021	0.0042	0.0018	0.0035	0.0035
0.25	0.0014	0.0028	0.0012	0.0023	0.0047	0.0047
0.30	0.0018	0.0036	0.0015	0.0030	0.0030	0.0012
0.35	0.0023	0.0046	0.0019	0.0038	0.0038	0.0015
0.40	0.0028	0.0012	0.0023	0.0047	0.0047	0.0019
0.45	0.0035	0.0014	0.0029	0.0029	0.0011	0.0023
0.50	0.0042	0.0018	0.0035	0.0035	0.0014	0.0028
(**d**)						
IR	Rt = 1	Rt = 2	Rt = 4	Rt = 8	Rt = 16	Rt = 32
0.01	0.0021	0.0042	0.0042	0.0017	0.0033	0.0014
0.05	0.0011	0.0022	0.0043	0.0018	0.0036	0.0036
0.10	0.0023	0.0046	0.0019	0.0038	0.0038	0.0015
0.15	0.0036	0.0015	0.0030	0.0030	0.0012	0.0024
0.20	0.0011	0.0021	0.0042	0.0042	0.0017	0.0034
0.25	0.0014	0.0028	0.0028	0.0011	0.0023	0.0045
0.30	0.0018	0.0036	0.0036	0.0015	0.0029	0.0012
0.35	0.0023	0.0046	0.0046	0.0018	0.0037	0.0015
0.40	0.0028	0.0028	0.0011	0.0023	0.0045	0.0019
0.45	0.0035	0.0035	0.0014	0.0028	0.0011	0.0023
0.50	0.0042	0.0042	0.0017	0.0034	0.0014	0.0028

Color key: Green: Expected infection after a negative preboarding test; Light yellow: Expected infection after negative arrival RPD test; Yellow: Expected infection after negative arrival RPD * test; Dark yellow: Expected infection after negative arrival PCR test; Light orange: Expected infection after negative arrival PCR test, quarantine for 4 days, and exit RPD test; Orange: Expected infection after negative arrival PCR test, quarantine for 4 days, and exit RPD * test; Dark orange: Expected infection after negative arrival PCR test, quarantine for 4 days, and exit PCR test; Red: Expected infection after negative arrival PCR test, quarantine for 4 days, and exit PCR plus RPD tests. Abbreviations: Expected transmission: the expected number of subsequent infections resulting from an individual. IR: Incidence rate; in fully vaccinated travelers, we used the breakthrough infection rate as the incidence rate. Rt: Effective reproduction number, the expected number of infections consequent to a single infected individual. PCR: Any test that meets the minimum requirement of sensitivity ≥ 98% and specificity ≥ 97%, usually achieved by Polymerase Chain Reaction test or other Nucleic Acid Amplification tests (NAAT). RPD *: Any test that meets the minimum requirement of sensitivity ≥ 90% and specificity ≥ 97%, can be achieved by many rapid antigen tests. RPD: Any test that meets the minimum requirement of sensitivity ≥ 80% and specificity ≥ 97% (WHO recommendation for COVID-19 diagnostic tests).

## Data Availability

Not applicable.
